# Canine tissue-associated CD4^+^CD8α^+^ double-positive T cells are an activated T cell subpopulation with heterogeneous functional potential

**DOI:** 10.1371/journal.pone.0213597

**Published:** 2019-03-13

**Authors:** Friederike V. Rabiger, Doris Bismarck, Martina Protschka, Gabriele Köhler, Peter F. Moore, Mathias Büttner, Heiner von Buttlar, Gottfried Alber, Maria Eschke

**Affiliations:** 1 Institute of Immunology/Molecular Pathogenesis, Center for Biotechnology and Biomedicine, College of Veterinary Medicine, University of Leipzig, Leipzig, Germany; 2 Institute of Pathology, Klinikum Fulda gAG, Fulda, Germany; 3 Department of Pathology, Microbiology and Immunology, School of Veterinary Medicine, University of California, Davis, Davis, United States; Instituto Nacional de Ciencias Medicas y Nutricion Salvador Zubiran, MEXICO

## Abstract

Canine CD4^+^CD8α^+^ double-positive (dp) T cells of peripheral blood are a unique effector memory T cell subpopulation characterized by an increased expression of activation markers in comparison with conventional CD4^+^ or CD8α^+^ single-positive (sp) T cells. In this study, we investigated CD4^+^CD8α^+^ dp T cells in secondary lymphatic organs (i.e. mesenteric and tracheobronchial lymph nodes, spleen, Peyer’s patches) and non-lymphatic tissues (i.e. lung and epithelium of the small intestine) within a homogeneous group of healthy Beagle dogs by multi-color flow cytometry. The aim of this systematic analysis was to identify the tissue-specific localization and characteristics of this distinct T cell subpopulation. Our results revealed a mature extrathymic CD1a^-^CD4^+^CD8α^+^ dp T cell population in all analyzed organs, with highest frequencies within Peyer’s patches. Constitutive expression of the activation marker CD25 is a feature of many CD4^+^CD8α^+^ dp T cells independent of their localization and points to an effector phenotype. A proportion of lymph node CD4^+^CD8α^+^ dp T cells is FoxP3^+^ indicating regulatory potential. Within the intestinal environment, the cytotoxic marker granzyme B is expressed by CD4^+^CD8α^+^ dp intraepithelial lymphocytes. In addition, a fraction of CD4^+^CD8α^+^ dp intraepithelial lymphocytes and of mesenteric lymph node CD4^+^CD8α^+^ dp T cells is TCRγδ^+^. However, the main T cell receptor of all tissue-associated CD4^+^CD8α^+^ dp T cells could be identified as TCRαβ. Interestingly, the majority of the CD4^+^CD8α^+^ dp T cell subpopulation expresses the unconventional CD8αα homodimer, in contrast to CD8α^+^ sp T cells, and CD4^+^CD8α^+^ dp thymocytes which are mainly CD8αβ^+^. The presented data provide the basis for a functional analysis of tissue-specific CD4^+^CD8α^+^ dp T cells to elucidate their role in health and disease of dogs.

## Introduction

Extrathymic CD4^+^CD8α^+^ double-positive (dp) T cells are a mature T cell subpopulation distinct from conventional CD4^+^ single-positive (sp) T helper and CD8α^+^ sp cytotoxic T cells known to occur in different species, e.g. swine, humans, monkeys, mice, rats, and chicken [1–9]. Canine CD4^+^CD8α^+^ dp T cells within peripheral blood mononuclear cells (PBMC) were first described around ten years ago [10–13]. To date, our group was able to characterize this unconventional T cell subpopulation in peripheral blood as mature T cell receptor (TCR) αβ^+^CD1a^-^ effector memory T cells [14,15]. CD4^+^CD8α^+^ dp T cells can develop from both, CD4^+^ sp as well as from CD8α^+^ sp T cells upon *in vitro* stimulation, but CD4^+^ sp T cells are the more potent progenitors [16]. Furthermore, canine CD4^+^CD8α^+^ dp T cells of the peripheral blood can be divided into three different subsets, i.e. CD4^dim^CD8α^bright^, CD4^bright^CD8α^bright^, and CD4^bright^CD8α^dim^, which differ in phenotype and functional features. The CD4^dim^CD8α^bright^ subset expresses the CD8αβ heterodimer, whereas most cells of the other two subsets express the unconventional CD8αα homodimer [14,15]. In contrast to CD8αβ, CD8αα does not work as a TCR co-receptor, but was shown to negatively regulate the activation of T cells [17].

Another unique feature of the total CD4^+^CD8α^+^ dp T cell subpopulation of PBMC is their significantly higher expression level of CD25 and of MHC-II in comparison to their CD4^+^ and CD8α^+^ sp counterparts, suggesting a high level of activation and an important immunological potential [15]. In fact, human CD4^+^CD8α^+^ dp T cells are associated with autoimmune diseases [18], infections, e.g. human immunodeficiency virus (HIV) infections [19], inflammatory bowel disease [20], atopic dermatitis [21], and breast cancer [22]. The latter are also common diseases of dogs, making the dog an interesting animal model for common human health disorders. Very recently, increased frequencies of CD4^+^CD8α^+^ dp T cells could be found in blood and spleen of dogs infected with *Ehrlichia chaffeensis* [23]. Additionally, CD4^+^CD8α^+^ dp T cells were reported to increase in the context of canine leishmaniasis [24], pointing to an important role during canine immune responses.

However, still the developmental origin and the function of canine CD4^+^CD8α^+^ dp T cells *in vivo* have not yet been clarified. For this purpose, reliable reference data in a homogeneous group of healthy animals including a comprehensive phenotypical and functional characterization not only from blood, but also from different lymphatic and non-lymphatic organs are required. Our group had the unique opportunity to study CD4^+^CD8α^+^ dp T cells of tracheobronchial (tLN) and mesenteric (mLN) lymph nodes, spleen, Peyer’s patches (PP), lung, and of small intestinal intraepithelial lymphocytes (IEL) from a standardized cohort of healthy Beagle dogs. As control, thymocytes of the same dogs were analyzed. The presented data allow a deeper insight into the characteristics of canine CD4^+^CD8α^+^ dp T cells of secondary lymphatic and mucosal organs. Besides, the data will provide the basis for further functional analyses to elucidate the *in vivo* role of CD4^+^CD8α^+^ dp T cells in dogs.

## Materials and methods

### Animals

Tissue samples were taken from healthy experimental Beagle dogs (Marshall Bioresources, North Rose, NY, USA, 12 dogs in total, six female, six male, age: 10–15 months) euthanized as control group of an animal experiment for preclinical drug development unrelated to our studies (approval number V54-19c 20/15-DA4/Anz.1004). All efforts were made to minimize suffering of the dogs. The physical health of all animals was confirmed by necropsy and histopathological examination. Directly after euthanasia, the dogs were fully bled and full thickness sections from duodenum and jejunum, mesenteric (mLN) and tracheobronchial (tLN) lymph nodes, lung, spleen, and thymus were collected for further processing (n = 10 for mLN, Peyer’s patches, lung, spleen, and thymus, n = 9 for tLN, n = 6 for intraepithelial lymphocytes of the small intestine).

### Generation of single cell suspensions of spleen, thymus and lymph nodes

1 x 1 x 0.5 cm^3^ pieces of spleen, thymic tissue, mLN, and tLN (size variable) were minced, passed through a 100 μm nylon cell strainer (BD Biosciences, Heidelberg, Germany) and resuspended in phosphate buffered saline (PBS). The cell suspensions were centrifuged at 500 x g for 10 min at 4°C. The splenic cells were treated with erythrocyte lysis buffer (150 mM NH_4_Cl, 8 mM KHCO_3_, 2 mM EDTA; pH 7) for 5 min at room temperature (RT) and the lysis reaction was stopped with PBS containing 3% fetal bovine serum (FBS, Thermo Fisher Scientific, Carlsbad, USA). Again, the splenic cell suspension was centrifuged at 500 x g for 10 min at 4°C and resuspended in PBS. The cell numbers were determined with a microscope using a hemocytometer (Laboroptik, Lancing, UK) and trypan blue (Sigma-Aldrich, Taufkirchen, Germany).

### Isolation of lung leukocytes

Tissue pieces of lung (3 x 3 x 0.5 cm^3^) were minced and digested for 30 min at 37°C in RPMI1640 medium (Biochrom, Berlin, Germany) supplemented with DNase I (111 U/ml; Sigma-Aldrich, Taufkirchen, Germany), Collagenase D (0.7 mg/ml; Roche Diagnostics Deutschland GmbH, Mannheim, Germany), and 1 mM sodium pyruvate (AppliChem, Darmstadt, Germany) with slow agitation. Single cell suspensions were obtained by passaging digested lung tissue through 100 μm cell strainers (BD Biosciences, Heidelberg, Germany). Subsequently, erythrocytes were lysed as described above. For enrichment of leukocytes, the cells were resuspended in 70% Percoll (GE Healthcare, Uppsala, Sweden) diluted in RPMI1640 medium and layered under 30% Percoll. Following density gradient centrifugation (400 x g, 20 min, RT), leukocytes were recovered from the interphase, resuspended in IMDM medium (PAN-Biotech, Aidenbach, Germany) supplemented with 100 U/ml penicillin, 100 μg/ml streptomycin (both purchased from PAA Laboratories), and 10% FBS (Thermo Fisher Scientific, Carlsbad, USA). The cell number was determined as described above.

### Isolation of lymphocytes from Peyer’s patches and from the small intestinal epithelium

A protocol to isolate lymphocytes from Peyer’s patches (PP) was adapted from a method described for pigs by Solano-Aguilar *et al*. [25]. Briefly, PP were collected from the duodenum and jejunum and immediately washed in ice-cold washing buffer consisting of Hank’s Balanced Salt Solution (HBSS) without Ca^2+^ and Mg^2+^ (PAN-Biotech, Aidenbach, Germany), 10 mM HEPES (Carl Roth, Karlsruhe, Germany) and 2% FBS (Thermo Fisher Scientific, Carlsbad, USA). After that, the intestinal epithelium was removed by incubating the pieces 3 x 30 min in washing buffer containing 2 mM 1,4-Dithioerythritol (DTE, Sigma-Aldrich, Taufkirchen, Germany), and 0.5 mM EDTA (Carl Roth, Karlsruhe, Germany) at 37°C under continuous stirring. The supernatant was discarded, whereas the tissue was minced with scalpels, dissociated by the gentleMACS Dissociator (Miltenyi Biotec, Bergisch Gladbach, Germany), and passaged through a 100 μm cell strainer (BD Biosciences, Heidelberg, Germany). Lymphocytes were separated from enterocytes by 40%/70% Percoll density gradient centrifugation (900 x g, 20 min, RT), harvested at the interphase, and washed in RPMI1640 medium (Biochrom, Berlin, Germany) containing 5% FBS and 50 μg/ml Gentamicin (Sigma-Aldrich, Taufkirchen, Germany).

For isolation of intraepithelial lymphocytes (IEL), duodenum and proximal jejunum were cut into 0.5 x 0.5 cm^2^ pieces under exclusion of PP and washed in ice-cold washing buffer (see above). The epithelium was separated from the lamina propria as described for PP. After the incubation process, the supernatant containing the epithelium was first filtered through a 100 μm cell strainer, and in a second step through a 70 μm cell strainer (SPL Life Sciences, Pocheon, South Korea). The mucus was removed by washing the cell pellet in a 25% Percoll solution (900 x g, 30 min, RT). Lymphocytes were separated from intestinal epithelial cells by 25%/47%/66% Percoll density gradient centrifugation (900 x g, 20 min, RT) and collected from the lymphocyte band. For both PP and IEL, erythrocytes were lysed and the cell number was determined as described above.

To confirm the appropriate separation of the epithelium from PP resp. the lamina propria, control samples before and after treatment were fixed in 4% formalin (Carl Roth, Karlsruhe, Germany) and embedded in paraffin (Leica, Heidelberg, Germany) followed by a hematoxylin-eosin stain (Merck, Darmstadt, Germany) and microscopical examination of 4 μm sections (S1 Fig).

### Flow cytometric analysis

To discriminate dead from viable cells, isolated cells from lymphatic and non-lymphatic organs were stained with fixable viability dye eFluor 780 (Thermo Fisher Scientific, Carlsbad, USA) according to the manufacturer’s protocol. Prior to direct labeling with fluorochrome-conjugated antibodies, the cells were incubated with a mixture of heat-inactivated normal serum derived from dog, rat, and mouse (each 15% in PBS) to block unspecific binding of Fc receptors. In case of indirect labeling with a secondary goat anti-mouse antibody, Fc receptor blockade was performed with a mixture of heat-inactivated rat, dog and goat normal serum (each 15% in PBS). Primary antibodies used for flow cytometric staining are summarized in Table 1. For definition of positive and negative populations during the analysis, fluorescence minus one (FMO) controls were included in the experiments. FMO controls contain all specific antibodies of the staining panel, except the one of interest which is replaced by its isotype control. The corresponding isotype control antibodies were purchased from Thermo Fisher Scientific (Carlsbad, USA) or Biolegend (San Diego, USA), respectively. The cells were incubated with primary antibodies for 15 min in the dark on ice. Canine CD1a, CD8β, TCRαβ, and TCRγδ were detected by the use of a PerCP/Cy5.5-conjugated goat-anti-mouse IgG secondary antibody (Biolegend, San Diego, USA). In case of exclusive surface staining, the cells were fixed with 2% paraformaldehyde (Sigma-Aldrich, Taufkirchen, Germany) for 15 min in the dark on ice prior to the analysis. For intracellular detection of granzyme B and FoxP3, the FoxP3/Transcription Factor Staining Buffer Set (Thermo Fisher Scientific, Carlsbad, USA) was used according to the manufacturer’s protocol. The cells were acquired with a BD LSR Fortessa flow cytometer (Becton Dickinson, Heidelberg, Germany) and viable CD5^+^ lymphocytes were analyzed using the FlowJo 10 software (Treestar Inc., Ashland, OR, USA) after doublet exclusion. See supporting information for gating strategy (S2 Fig).

**Table 1:** Primary antibodies used for flow cytometry.

Antigen	Clone	Species Reactivity	Isotype	Formats
CD5	YKIX322.3^a^	dog	Rat IgG2a	PE PerCP-eFluor 710
CD4	YKIX302.9^a^^,^ ^b^	dog	Rat IgG2a	FITC, APC Pacific Blue
CD8α	YCATE55.9^a^^,^ ^b^	dog	Rat IgG1	APC Pacific Blue Alexa Fluor 647
CD1a	CA13.9H11^c^	dog	Mouse IgG1	Hybridoma supernatant
CD8β	CA15.4G2^c^	dog	Mouse IgG1	Hybridoma supernatant Biotin
TCRγδ	CA20.8H1^c^	dog	Mouse IgG1	Hybridoma supernatant
TCRαβ	CA15.8G7^c^	dog	Mouse IgG1	Hybridoma supernatant
CD25	P4A10^a^	dog	Mouse IgG1	PE
FoxP3	FJK-16s^a^	mouse/rat published crossreactivity with dog [14,26,27]	Rat IgG2a	FITC
Granzyme B	GB11^d^	human/mouse published crossreactivity with dog [14]	Mouse IgG1	FITC

^a^ Thermo Fisher Scientific, Carlsbad, USA

^b^ Bio-Rad, Munich, Germany

^c^ Leukocyte Antigen Biology Laboratory, VM PMI, School of Veterinary Medicine, University of California, Davis, USA

^d^ Biolegend, San Diego, USA

### Statistical analysis

The evaluation of statistical significance was performed using Graph Pad Prism 5.01 software (San Diego, CA, USA). Kolmogorov-Smirnov test (with Dallal-Wilkinson-Lilliefor p value) was applied to test for normality. In case of normal distribution of all data sets mean values are presented within one graph. The unpaired Student’s t-test (two-tailed) was used to compare two groups whereas differences between more than two groups were analyzed by One-way analysis of variance (ANOVA) with Bonferroni post hoc test. If graphs include nonparametric data, the median is depicted. Multiple comparisons were performed by use of the Kruskal-Wallis H test with Dunn’s post test. If only two groups were included in the analysis, the Mann-Whitney U test (two-tailed) was used. The level of confidence for significance is depicted in figure legends.

## Results

### Highest frequencies of mature CD4^+^CD8α^+^ double-positive T cells are present in Peyer’s patches compared to other secondary lymphatic and non-lymphatic organs of healthy dogs

**Fig 1:**
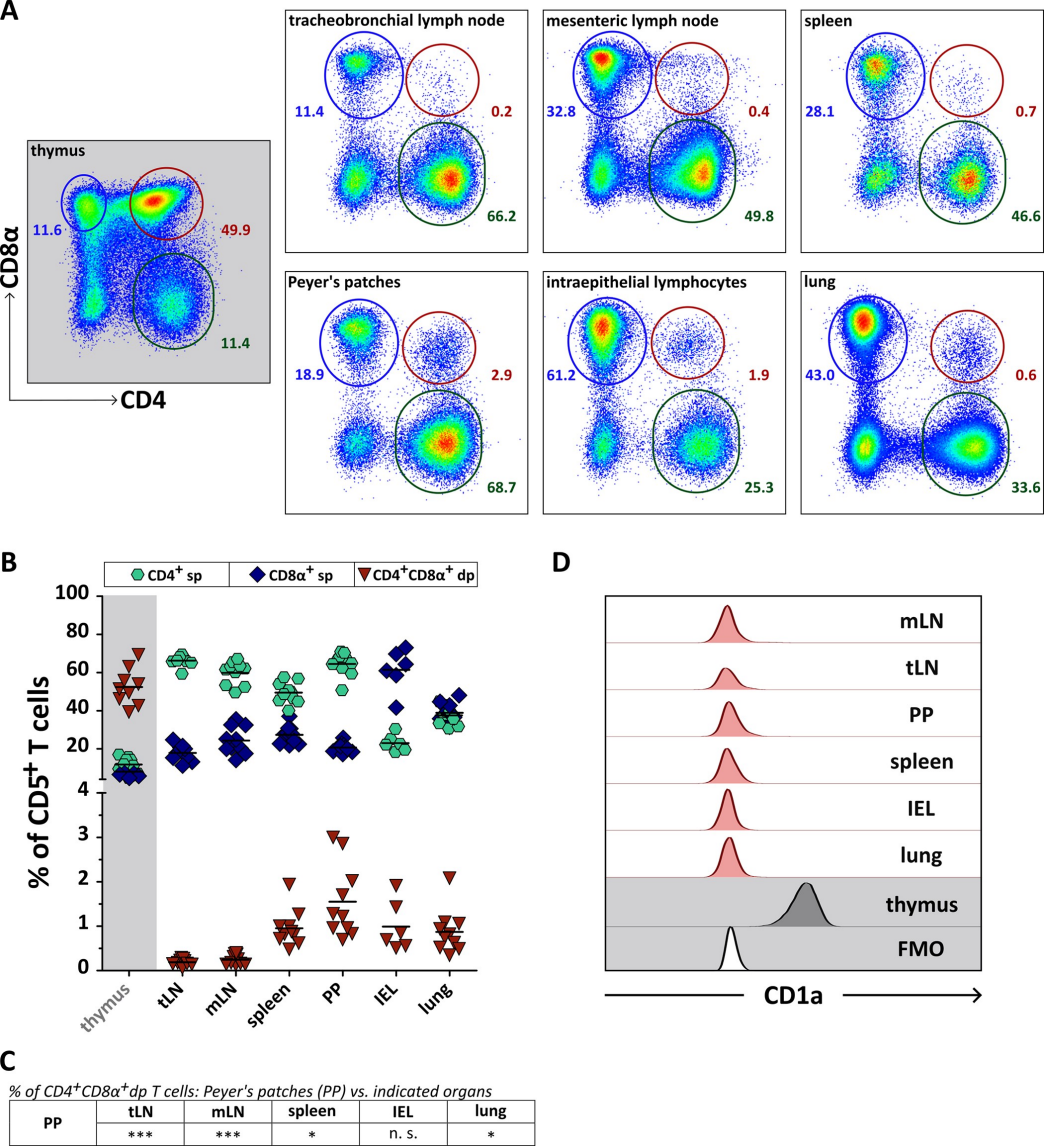
Mature CD4^+^CD8α^+^ double-positive (dp) T cells are present in secondary lymphatic organs as well as in non-lymphatic tissues of healthy dogs with highest frequencies in Peyer’s patches. (A) The frequency of canine CD4^+^ single-positive (sp), CD8α^+^ sp, and CD4^+^CD8α^+^ dp T cells in different organs was analyzed by flow cytometry. Shown are representative pseudocolor plots with numbers indicating percentages. Organs of 6-10 dogs in total have been analyzed in two independent experiments. Thymus was used as control (grey background). Only living non-doublet CD5^+^ T cells were included in the analysis. The gating strategy is shown in supporting S2 Fig. (B) Quantification of CD4^+^ sp (green hexagons), CD8α^+^ sp (blue diamonds), and CD4^+^CD8α^+^ dp (red triangles) T cells in canine tracheobronchial lymph nodes (tLN), mesenteric lymph nodes (mLN), spleen, Peyer’s patches (PP), intraepithelial lymphocytes of the small intestine (IEL), and lung is depicted. Thymus served as control (grey background). Pooled data of two independent experiments are shown. Each dot represents one individual dog, the horizontal bars indicate mean values. For clarity reasons, a statistical analysis (One-way ANOVA with Bonferroni’s Multiple Comparison Test, * p < 0.05, *** p < 0.001, n. s.: not significant) is shown in (C). (D) Tissue-associated canine CD4^+^CD8α^+^ dp T cells are mature T cells lacking the thymic marker CD1a (red histograms). As control, thymus (grey histogram) was analyzed for CD1a expression with the appropriate fluorescence minus one (FMO) control (white histogram). Results of one representative dog are depicted. Organs of 4–10 dogs in total have been analyzed in two independent experiments.

To gain an in depth understanding of canine CD4^+^CD8α^+^ double-positive (dp) T cells, a further characterization in other tissues than PBMC is required. Here we analyzed different lymphatic and non-lymphatic organs, i.e. tracheobronchial (tLN) and mesenteric (mLN) lymph nodes, spleen, Peyer’s patches (PP), lung, and small intestinal intraepithelial lymphocytes (IEL) from a homogeneous cohort of healthy Beagle dogs. In all analyzed organs a fraction of CD4^+^CD8α^+^ dp T cells could be detected (Fig 1A). Our analysis revealed significant differences concerning the CD4^+^CD8α^+^ dp T cell frequencies depending on the organ, with highest frequencies in PP (1.6% on average) and lowest in tLN (0.2% on average) (Fig 1B and 1C). It is known that canine CD4^+^CD8α^+^ dp T cells of the peripheral blood form a heterogeneous cell population which can be divided into three different subsets, namely CD4^bright^CD8α^dim^, CD4^bright^CD8α^bright^, and CD4^dim^CD8α^bright^ [15]. In contrast to PBMC, the CD4^+^CD8α^+^ dp T cells of spleen, PP, IEL and lung constitute one homogeneous population which cannot be divided into different subsets. In tLN and mLN a more heterogeneous, but very small population of CD4^+^CD8α^+^ dp T cells lacking the CD4^bright^CD8α^bright^ subset is present (Fig 1A). Yet a subdivision into subsets would have restricted further analyses due to low cellular numbers. As expected, the CD4^+^CD8α^+^ dp fraction in the thymus serving as control is predominating (Fig 1B). These immature thymocytes, however, express the thymic marker CD1a, which distinguishes them from the CD1a^-^CD4^+^CD8α^+^ dp T cells in secondary lymphatic organs, lung and intestine (Fig 1D) and from previously reported peripheral blood CD1a^-^CD4^+^CD8α^+^ dp T cells [15]. Consequently, the extrathymic CD4^+^CD8α^+^ dp T cells exhibit a mature phenotype.

### CD4^+^CD8α^+^ double-positive T cells of secondary lymphatic and non-lymphatic organs differ from CD8α^+^ single-positive (sp) T cells and CD4^+^CD8α^+^ dp thymocytes in their CD8αα expression, and a high proportion is TCRαβ^+^

**Fig 2:**
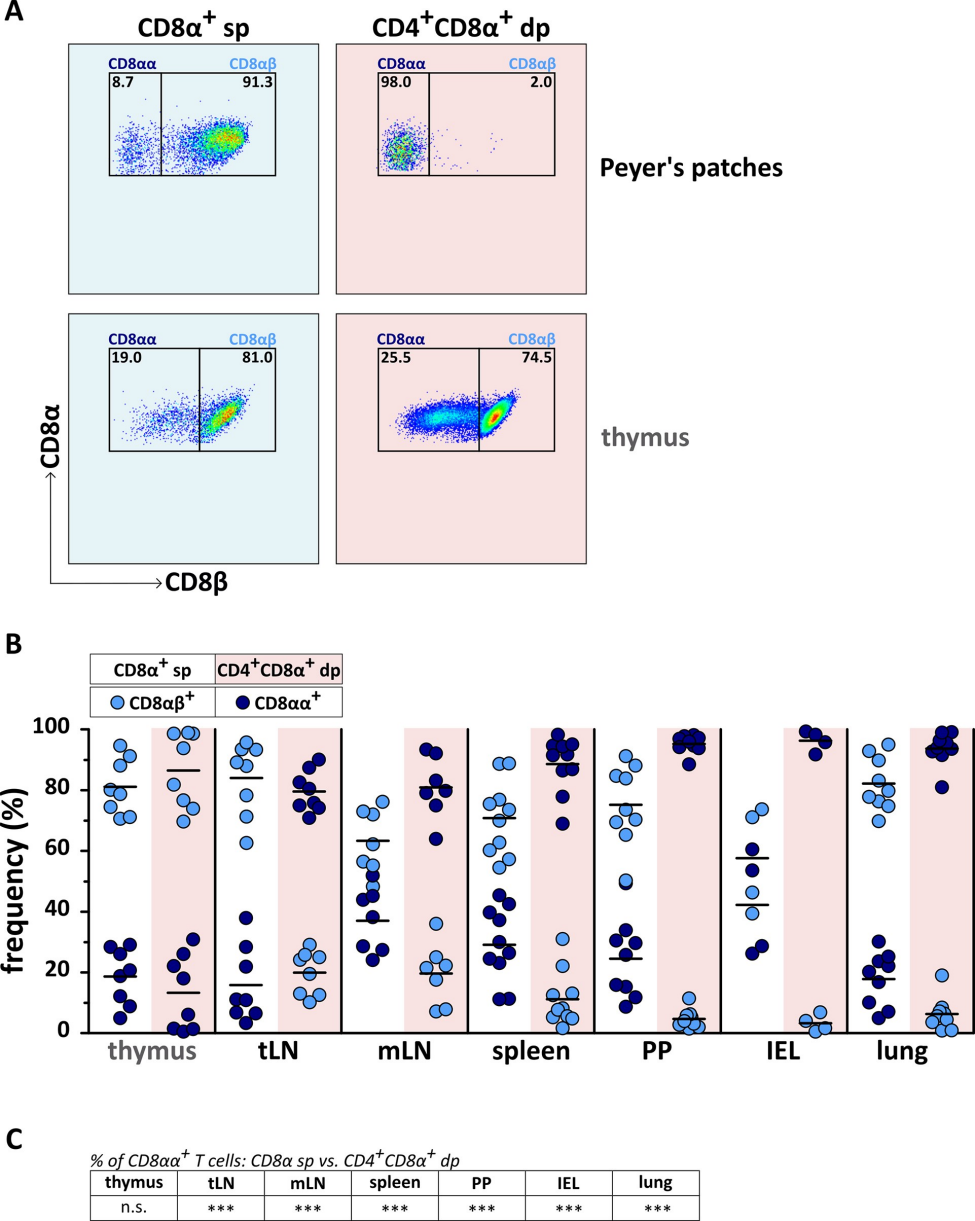
The majority of canine CD4^+^CD8α^+^ double-positive (dp) T cells in secondary lymphatic and non-lymphatic organs is characterized by expression of the CD8αα homodimer. (A) CD4^+^CD8α^+^ dp T cells were analyzed by flow cytometry for the expression of CD8αα and CD8αβ in comparison to CD8α^+^ single-positive (sp) T cells. The pseudocolor plots of Peyer’s patches stand representative for all secondary lymphatic and non-lymphatic organs in this study. Thymocytes are shown for control. The numbers in the pseudocolor plots imply percentages. Organs of 4–10 dogs in total have been analyzed in two independent experiments. The CD8α^+^CD8β^-^ population indicates a CD8αα homodimer, the CD8α^+^CD8β^+^ population a CD8αβ heterodimer. (B) Proportions of CD8αα (dark blue dots) vs. CD8αβ (light blue dots) expression of CD8α^+^ sp (white background) and mature CD4^+^CD8α^+^ dp T cells (red background) in tracheobronchial lymph node (tLN), mesenteric lymph node (mLN), spleen, Peyer’s patches (PP), intraepithelial lymphocytes of the small intestine (IEL), and lung with thymus as control were quantified. Pooled data of two independent experiments are shown. Each dot represents one individual dog, the horizontal bars indicate mean values. Differences in percentages of CD8αα^+^ cells among CD4^+^CD8α^+^ dp and CD8α^+^ sp T cells were analyzed by unpaired Student’s t-test (two-tailed; *** p < 0.001) and are summarized in (C).

CD8^+^ T cells express their CD8 surface receptor either as a CD8αβ heterodimer or as a CD8αα homodimer. CD8αβ promotes the activation of T cells with low-affinity TCR, whereas CD8αα increases the activation threshold [17,28]. Therefore, we investigated the composition of the CD8 receptor of mature CD4^+^CD8α^+^ dp extrathymic T cells and compared it with their CD8**α**^+^ sp counterparts, and with immature CD4^+^CD8**α**^+^ dp thymocytes. In all analyzed organs, as expected, the majority of the CD8**α**^+^ sp subpopulation is CD8β^+^ indicating expression of the CD8αβ heterodimer (Fig 2A and 2B). As in blood, only a small fraction of CD8α^+^ sp T cells does not express CD8β, equivalent with a CD8αα phenotype. In the thymus, both the CD8α^+^ sp and the CD4^+^CD8α^+^ dp fraction are predominantly CD8αβ^+^. In marked contrast to this, the main CD8 receptor of the mature extrathymic CD4^+^CD8α^+^ dp T cells is the CD8αα homodimer, whereas the CD8αβ heterodimer only comprises a small proportion (Fig 2A–2C), underlining the distinct features of this T cell subpopulation.

**Fig 3:**
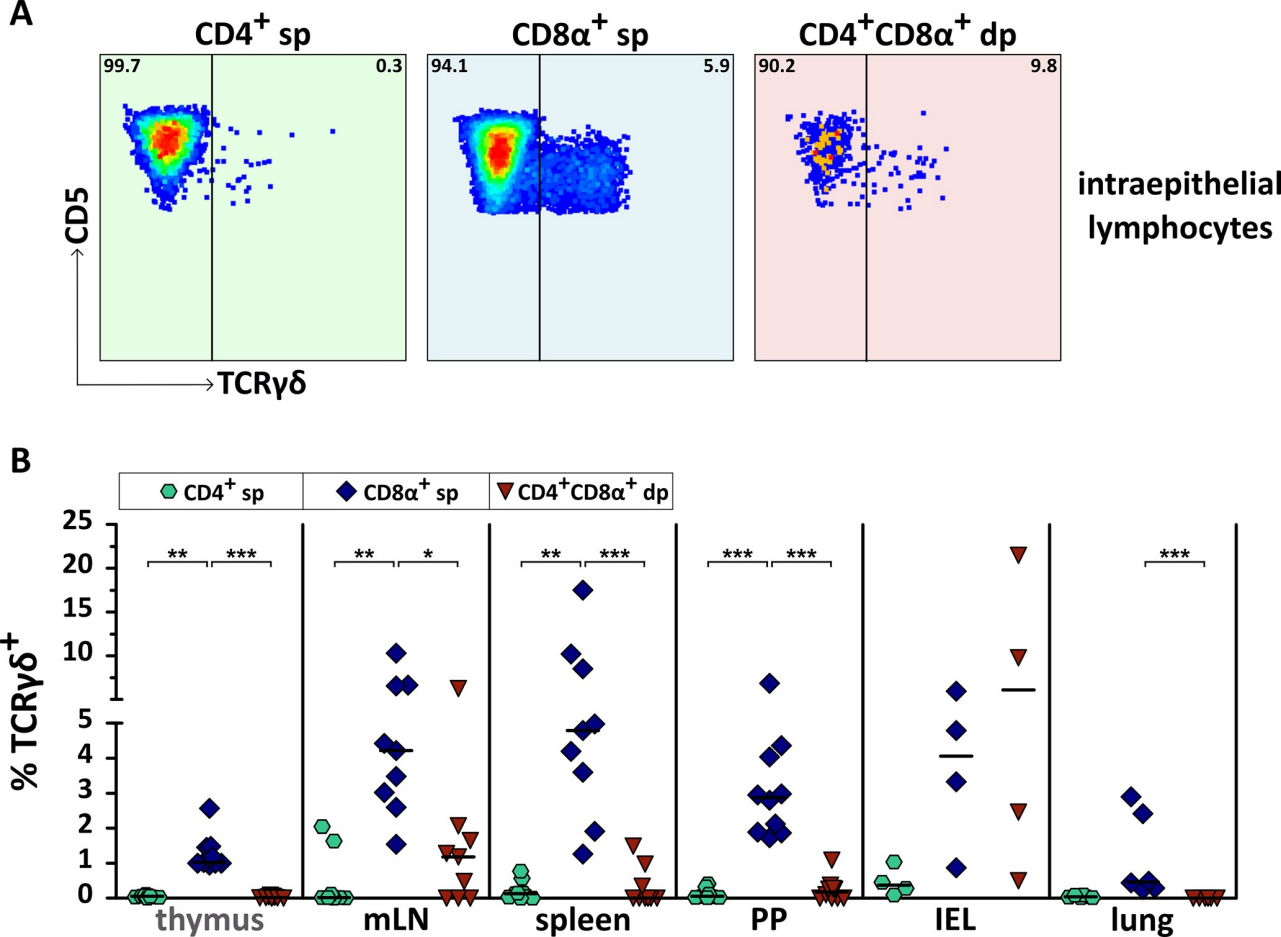
Canine CD4^+^CD8α^+^ double-positive (dp) T cells in secondary lymphatic and non-lymphatic organs are mainly TCRγδ-negative. (A) Shown are representative results of T cell receptor γδ (TCRγδ) expression in CD4^+^CD8α^+^ dp, CD4^+^ sp, and CD8α^+^ sp T cells among intraepithelial lymphocytes of the small intestine (IEL). The numbers in the flow cytometry plots indicate percentages. (B) Quantification of the frequency of TCRγδ^+^ cells among CD4^+^ sp (green hexagons), CD8α^+^ sp (blue diamonds), and CD4^+^CD8α^+^ dp (red triangles) T cells in mesenteric lymph node (mLN), spleen, Peyer’s patches (PP), IEL, and lung is shown. Thymus is depicted for control. Pooled data of two independent experiments are shown. Each dot represents one individual dog, the horizontal bars indicate median values. Statistical analysis was performed by One-way ANOVA with Dunn’s Multiple Comparison Test (* p < 0.05, ** p < 0.01, *** p < 0.001).

As one subpopulation of unconventional CD8αα^+^ sp T cells of IEL from small intestine of dogs has been published to express the T cell receptor γδ (TCRγδ) [29], we were interested in the TCRγδ expression of CD4^+^CD8α^+^ dp extrathymic T cells in peripheral tissues. Despite their CD4^+^CD8αα^+^ phenotype, TCRγδ^+^ dp T cells are nearly absent in lung, spleen, mLN, PP, and thymus (Fig 3A). This result pointed to the expression of TCRαβ and was confirmed by direct TCRαβ staining of splenic CD4^+^CD8α^+^ dp T cells of the same dogs (S3 Fig). Due to limited cell number, tLN could not be included in TCRγδ analysis. Interestingly, although with a high inter-individual variation, only within IEL (median ~6%) and mLN (median ~1%) a proportion of CD4^+^CD8α^+^ dp T cells was found to be TCRγδ^+^ (Fig 3A and 3B). This is in contrast to CD8α^+^ sp T cells from which up to 5% express TCRγδ in all analyzed organs (Fig 3B).

### A high proportion of tissue-associated mature CD4^+^CD8α^+^ dp T cells expresses the activation marker CD25

**Fig 4:**
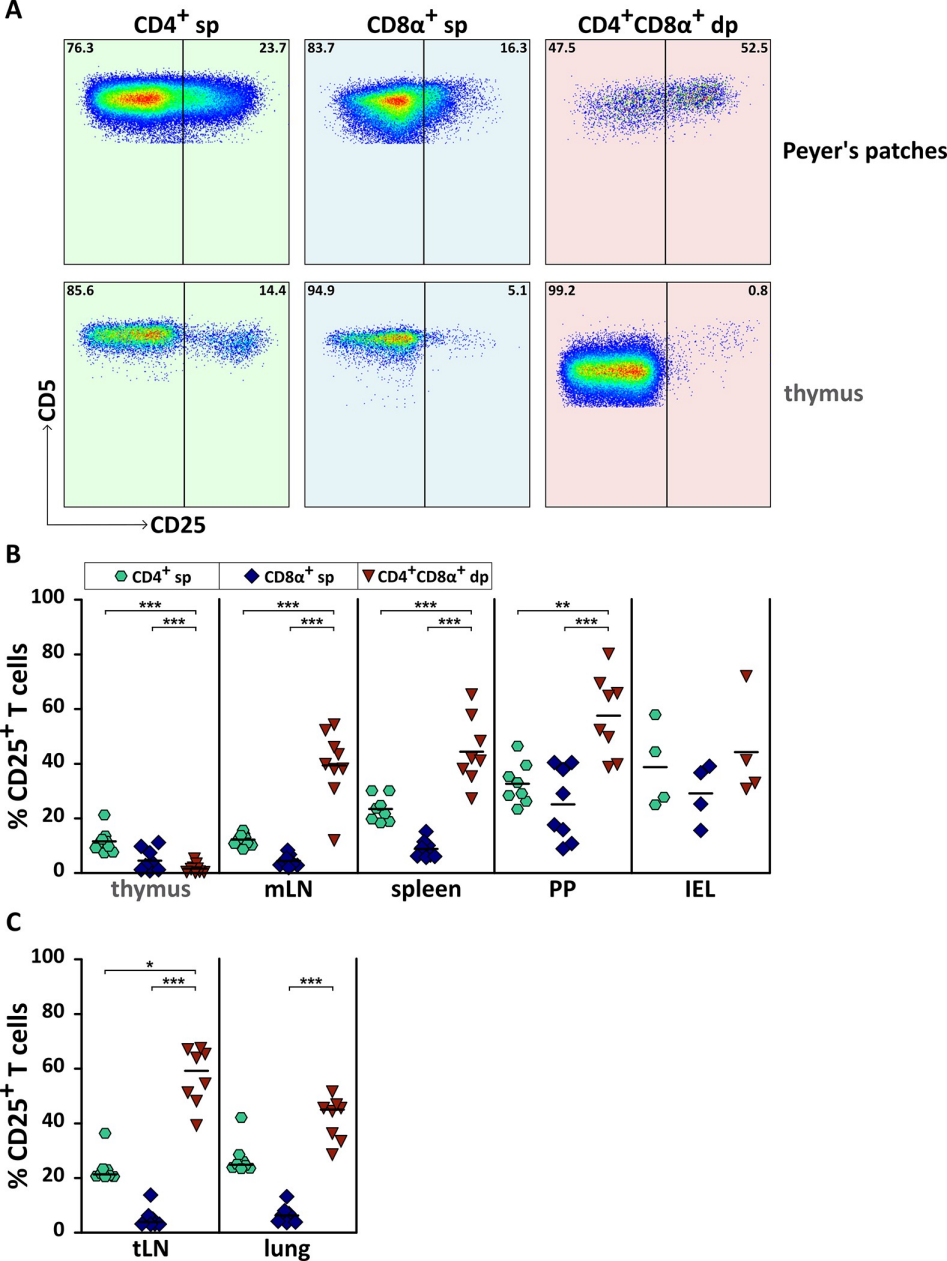
Canine CD4^+^CD8α^+^ double-positive (dp) T cells of secondary lymphatic and non-lymphatic organs constitutively express CD25 in contrast to CD4^+^CD8α^+^ dp thymocytes. (A) The expression of CD25 on CD4^+^ single-positive (sp), CD8α^+^ sp and CD4^+^CD8α^+^ dp T cells was analyzed by flow cytometry. Shown are representative data of Peyer’s patches in comparison to thymus. The numbers in the pseudocolor plots indicate percentages. Two independent experiments have been performed including organs of 4–10 dogs in total. (B + C) Pooled data of CD25 expression on CD4^+^ sp (green hexagons), CD8α^+^ sp (blue diamonds), and CD4^+^CD8α^+^ dp (red triangles) T cells in indicated organs are depicted. (B) The graphs include normally distributed data values, each symbol represents one individual dog. The horizontal bars indicate mean values. Statistical analysis was performed by One-way ANOVA with Bonferroni’s Multiple Comparison Test (** p < 0.01, *** p < 0.001). (C) The graphs show non-normally distributed data values, each symbol represents one individual dog. The horizontal bars indicate median values. Statistical analysis was performed by One-way ANOVA with Dunn’s Multiple Comparison Test (* p < 0.05, *** p < 0.001).

CD4^+^CD8α^+^ dp thymocytes are known as an immature intermediate stage during T cell development. Accordingly, these cells lack expression of activation markers such as CD25 [30–32], which we could confirm in our study (Fig 4A and 4B). In contrast, mature CD4^+^CD8α^+^ dp T cells of secondary lymphatic and non-lymphatic tissues constitutively show high frequencies of CD25 expression (Fig 4A–4C), in line with our findings about CD4^+^CD8α^+^ dp T cells of the peripheral blood [14,15] and corresponding to an activated phenotype. Compared to their single-positive counterparts, frequencies of CD25^+^ T cells among CD4^+^CD8α^+^ dp T cells were significantly higher in all analyzed secondary lymphatic organs (tLN, mLN, spleen, PP). Interestingly, despite the high abundance of CD25^+^ T cells within the CD4^+^CD8α^+^ dp subpopulation, the IEL of the small intestine revealed no significant difference between CD4^+^ or CD8α^+^ sp and CD4^+^CD8α^+^ dp T cells concerning their CD25 expression (Fig 4B).

### CD4^+^CD8α^+^ dp T cells in lymph nodes contain FoxP3^+^ regulatory T cells

**Fig 5:**
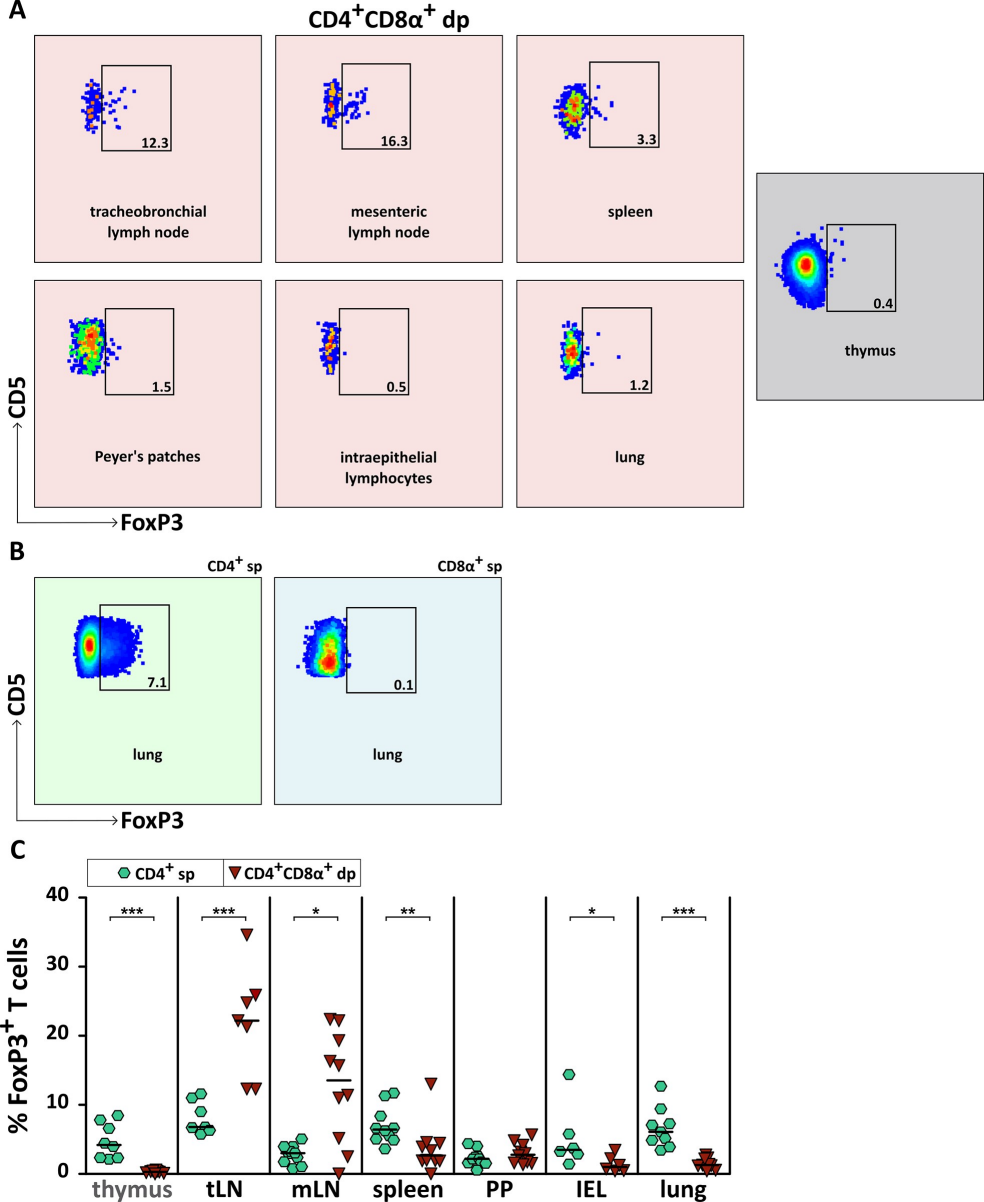
A proportion of lymph node CD4^+^CD8α^+^ double-positive (dp) T cells comprises FoxP3^+^ regulatory T cells. (A) Representative results of FoxP3 expression in CD4^+^CD8α^+^ dp T cells of secondary lymphatic and non-lymphatic organs, and of CD4^+^CD8α^+^ dp thymocytes are shown. Two independent experiments with n = 4–10 dogs in total were performed. The numbers in the flow cytometry plots indicate percentages. Appropriate gating was confirmed by internal negative (CD8α^+^ single-positive (sp) T cells) and positive (CD4^+^ sp T cells) controls (B). Presented is the FoxP3 expression of CD4^+^ sp and CD8α^+^ sp T cells in canine lung being representative for all analyzed organs. (C) Proportions of FoxP3 expression of CD4^+^ sp (green hexagons) and CD4^+^CD8α^+^ dp (red triangles) T cells were analyzed in indicated organs (tLN: tracheobronchial lymph node, mLN: mesenteric lymph node, PP: Peyer’s patches, IEL: intraepithelial lymphocytes of the small intestine). Each symbol represents one individual dog, the horizontal bars indicate median values. The Mann-Whitney U test was performed to check for statistical significance between CD4^+^ sp and CD4^+^CD8α^+^ dp T cells (two-tailed; * p < 0.05, ** p < 0.01, *** p < 0.001).

Given the high frequencies of CD25 in dp T cells, we analyzed the transcription factor forkhead box P3 (FoxP3) which is specific for regulatory T cells (Treg) [33]. As expected, immature CD4^+^CD8α^+^ dp thymocytes are FoxP3^-^ (Fig 5A and 5C). FoxP3 expression of mature CD4^+^CD8α^+^ dp T cells of secondary lymphatic and non-lymphatic organs is heterogeneous. In all analyzed organs except lymph nodes, only low proportions of CD4^+^CD8α^+^FoxP3^+^ dp T cells (≤ 3%) were detected. In lymph nodes, however, percentages reach ~10% in mLN and ~20% in tLN. Furthermore, these percentages are even significantly higher than are those of mLN and tLN CD4^+^FoxP3^+^ sp T cells (Fig 5C). Although the CD4^+^CD8α^+^ dp T cell subpopulation in lymph nodes is rather small (Fig 1), the distribution of fluorescence (i.e. mean fluorescence intensity, MFI) of the FoxP3^+^ events is comparable to the positive control (i. e. CD4^+^ sp T cells), indicating a valid signal [34]. Furthermore, the intracellular expression of FoxP3 in lymph nodes correlates with the degree of surface expression of CD25 on CD4^+^CD8α^+^ dp T cells (S4 Fig). Taken together, these results point to a regulatory potential of CD4^+^CD8α^+^ dp T cells in lymph nodes.

### IEL CD4^+^CD8α^+^ dp T cells express the cytotoxicity marker granzyme B

**Fig 6:**
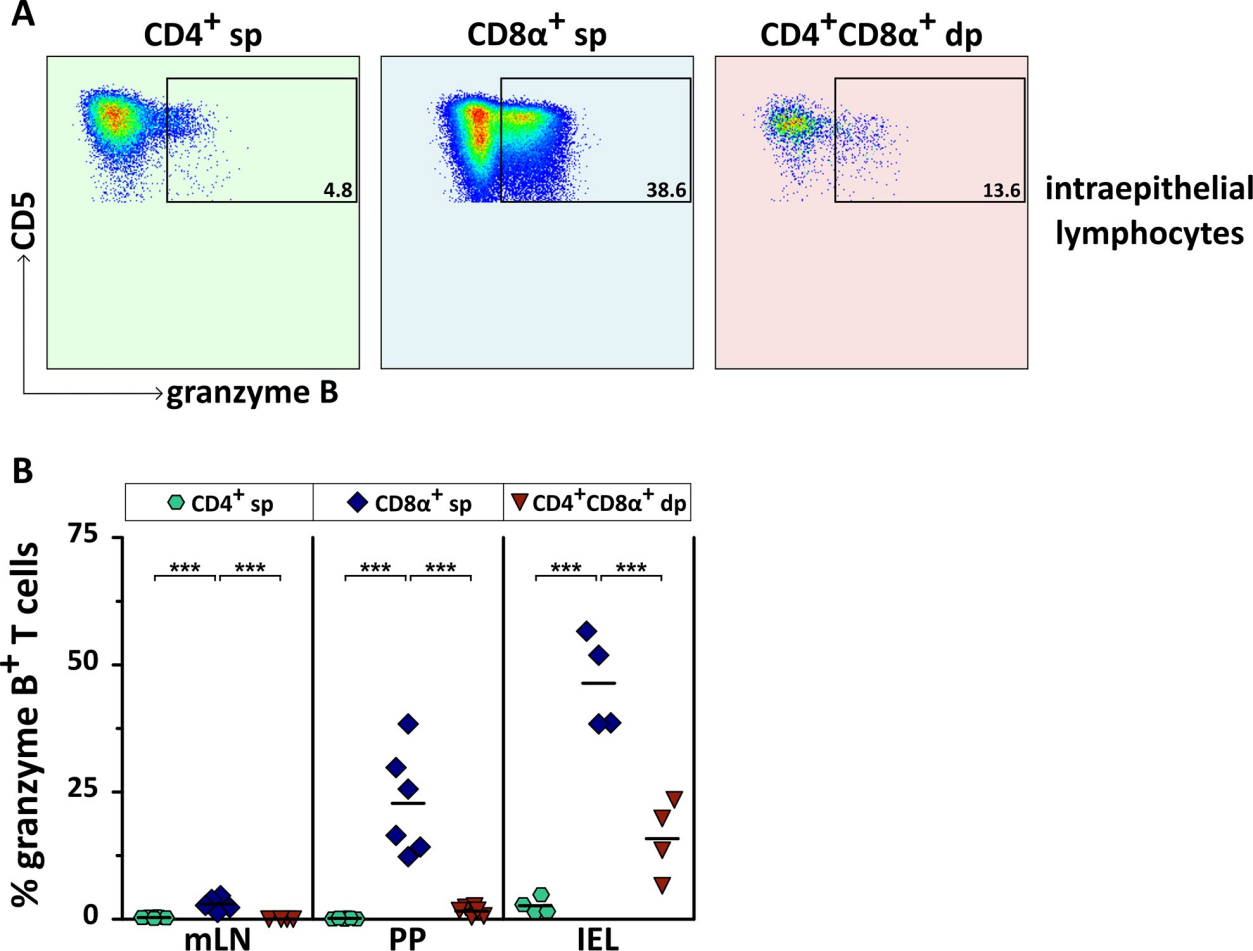
CD4^+^CD8α^+^ double-positive (dp) T cells of the small intestinal epithelium express the cytotoxic molecule granzyme B. (A) Representative results of granzyme B expression in CD4^+^CD8α^+^ dp T cells of small intestinal intraepithelial lymphocytes (IEL) are shown in comparison to CD4^+^ single-positive (sp) and CD8α^+^ sp T cells. The numbers in the flow cytometry plots indicate percentages. (B) Quantification of the frequency of granzyme B^+^ cells among CD4^+^ sp (green hexagons), CD8α^+^ sp (blue diamonds), and CD4^+^CD8α^+^ dp (red triangles) T cells in mesenteric lymph node (mLN), Peyer’s patches (PP) and IEL is depicted. Each symbol represents one individual dog, the horizontal bars indicate mean values. Statistical analysis was performed by One-way analysis of variance (ANOVA) with Bonferroni post hoc test (*** p < 0.001).

Finally, we investigated whether CD4^+^CD8α^+^ dp T cells of lymphatic and non-lymphatic tissues show features of cytotoxic T cells by analyzing their intracellular granzyme B expression. As most IEL are known to exhibit constitutive cytolytic capacity in human and mice [28,35], we focused on the intestinal environment. While granzyme B expression in CD4^+^CD8α^+^ dp T cells of gut-associated lymphoid tissue (GALT) is absent (mLN 0%) resp. low (PP 1.6% on average), a considerable proportion (15% on average) of IEL CD4^+^CD8α^+^ dp T cells is granzyme B^+^ (Fig 6). In accordance with literature [36,37], some CD4^+^ sp T cells expressing granzyme B are also present in the small intestinal epithelium. As expected, a high proportion of CD8α^+^ sp T cells expresses granzyme B within IEL. Furthermore, granzyme B^+^ CD8α^+^ sp T cells are present in PP but not in mLN.

## Discussion

Canine CD4^+^CD8α^+^ double-positive (dp) T cells of peripheral blood are a heterogeneous effector memory T cell subpopulation suggesting an important role in immune response by their constitutively high activation [14,15]. Here we provide the first systematic characterization of CD4^+^CD8α^+^ dp T cells in different tissues within one homogeneous group of healthy Beagle dogs. This reveals insight into potential induction and/or effector sites of this unique T cell subpopulation and is a prerequisite for subsequent functional analyses. Our comprehensive study shows that highest frequencies of CD4^+^CD8α^+^ dp T cells are present in Peyer’s patches (PP) compared to tracheobronchial and mesenteric lymph nodes (LN), spleen, intraepithelial lymphocytes of the small intestine (IEL), and lung. Former reports about canine CD4^+^CD8α^+^ dp T cells in LN [24,38], bone marrow [24], spleen [23], and IEL [39] are limited by either a low number [23] or by heterogeneity in breed, age, and/or health status of dogs [24,38,39]. Moreover, a detailed characterization of these cells was still missing. We could demonstrate that the majority of CD4^+^CD8α^+^ dp T cells in dogs is TCRαβ^+^, and, interestingly, up to 96% on average (IEL, PP) express the unconventional CD8αα homodimer. CD8αα was formerly thought to be a functional homolog of the MHC class I binding TCR co-receptor CD8αβ [40]. However, the CD8β chain has been identified as key molecule for CD8-dependent TCR function [41]. Transient co-expression of CD8αα on CD8αβ^+^ T cells, in contrast, results in decrease of TCR signal transduction inducing a higher activation threshold proportional to the expression level of CD8αα. Nevertheless, increased antigen stimulation can overcome the CD8αα repressor function [17,42]. CD4^+^CD8α^+^ dp T cells are characterized by high frequencies of CD25 expression, the IL-2 receptor α-chain (IL-2Rα) which constitutes the high-affinity IL-2 receptor in combination with IL-2Rβ and IL-2Rγ. The high-affinity IL-2 receptor enables rapid and effective proliferation of T cells [43]. As CD8αα has been shown to repress T cell activation [17,42], it might thus contribute to adequate induction of CD4^+^CD8α^+^ dp T cell effector functions only upon high antigenic stimulation and thereby prevent excessive immune responses. However, the function of CD8αα on CD4^+^ single-positive (sp) T cells has not yet been clarified and needs further investigation.

As demonstrated in previous *in vitro* studies, based on oligoclonal resp. polyclonal stimulation, canine PBMC CD4^+^CD8α^+^ dp T cells can develop from both, CD8α^+^ and CD4^+^ sp T cells, the latter being the more potent progenitors. CD4^+^CD8α^+^ dp T cells emerging from CD4^+^ sp T cells express CD8αα, whereas CD4^+^CD8α^+^ dp T cells developing from CD8α^+^ sp T cells express CD8αα or CD8αβ [16]. Thus, the predominance of the CD8αα homodimer might be related to the main origin from CD4^+^ sp T cells. In dogs, mice and humans the CD8αα isoform is constitutively expressed by TCRαβ^+^ and TCRγδ^+^CD8^+^ sp IEL [28,29,35,44,45] In addition, CD8αα can be upregulated on murine TCRαβ^+^CD4^+^ IEL *in vivo* [46,47], and *in vitro* in the presence of TGF-β and retinoic acid (RA) [36,48]. Furthermore, RA is produced by dendritic cells of the gut-associated lymphoid tissue (GALT), including PP [49,50]. This, in combination with high antigenic exposure in the gut, might lead to the induction of more CD4^+^CD8αα^+^ dp T cells in PP and in the intestinal epithelium than in LN, where RA is also present [51], but the antigenic load and diversity are less abundant. A possible association with antigenic stimulation was also hypothesized for porcine dp T cells [2] that account for a larger proportion of total CD4^+^ lymphocytes in mucosa-associated lymphoid tissues as compared to lymph nodes [3,52]. In mice, many CD4^+^ IEL co-express CD8α [48,53], whereas CD4^+^CD8α dp T cells are rare in secondary lymphatic organs [1,8,53,54]. In humans, a significant percentage of CD4^+^CD8α^+^ dp T cells has been described in the lamina propria of the gut [55]. Overall, this tissue distribution is related to an immunoregulatory and/or immunosurveillance function of CD4^+^CD8α^+^ dp T cells as suggested previously [2].

Interestingly, we found evidence that the expression pattern of functional markers on canine CD4^+^CD8α^+^ dp T cells is tissue-dependent, indicating heterogeneous functional potential. We show that CD4^+^CD8α^+^ dp T cells of spleen, PP, IEL, and lung are mainly FoxP3^-^. However, this transcription factor specific for regulatory T cells reaches significant proportions within LN. In contrast, comparable numbers of FoxP3^+^CD4^+^CD8α^+^ dp T cells with regulatory potential are present in mLN, spleen, and thymus of pigs [56]. According to van Kaer *et al*., murine CD4^+^CD8α^+^ T cells induced *in vitro* only transiently express FoxP3 [57]. In support of this, Sujino *et al*. could demonstrate *in vivo* that murine regulatory T cells of the intestinal *lamina propria* lose FoxP3 expression upon migration into the intestinal epithelium where they become FoxP3^-^ regulatory CD4^+^CD8αα^+^ IEL [58]. Furthermore, human CD4^+^CD8αα^+^ dp T cells of the colonic lamina propria have been shown to represent a regulatory T cell subset despite the lack of FoxP3 expression [20]. This leads us to the hypothesis that, in addition to FoxP3^+^CD4^+^CD8α^+^ dp T cells in canine LN, FoxP3^-^CD4^+^CD8α^+^ dp T cells might possess regulatory properties. However, further experiments are necessary to verify this hypothesis. In addition to regulatory CD4^+^CD8α^+^ dp IEL [58], murine CD4^+^CD8α^+^ dp IEL with cytolytic activity [36] have been described indicating the presence of different functional subsets. Since we found evidence by granzyme B staining that canine IEL CD4^+^CD8α^+^ dp T cells also partly exhibit cytotoxic potential, the spectrum of effector functions of these cells should be subject of further research.

Taken together, this study provides a comprehensive characterization of CD4^+^CD8α^+^ dp T cells in lymphatic and non-lymphatic organs of a homogeneous cohort of healthy Beagle dogs. We define these tissue-associated CD4^+^CD8α^+^ dp T cells as an activated, but possibly self-regulating (by CD8αα expression) T cell subpopulation with heterogeneous functional (i.e. regulatory or cytotoxic) potential depending on its localization. This lays the foundation for future work on the role of CD4^+^CD8α^+^ dp T cells in various organ-specific diseases of dogs.

## Supporting information

S1 Fig The separation of the epithelium from Peyer’s patches and small intestinal villi was confirmed by hematoxylin and eosin (H&E) staining before and after treatment with DTE/EDTA. Representative H&E-stained sections of Peyer’s patches (A) and small intestine (B) before DTE/EDTA treatment are shown. Normal villous architecture with intact epithelium is visible. After DTE/EDTA treatment, the epithelial layer is removed from Peyer’s patches (C), and from the villi (D), respectively. For the latter this is displayed at higher magnifications in the insets (compare B + D).
(TIF)

S2 Fig General gating strategy used in flow cytometric analyses. Representative pseudocolor plots of Peyer’s patches are depicted to show the general gating strategy. After exclusion of dead cells (A), gating on lymphocytes was performed according to their forward and side scattering (FSC/SSC) properties (B). Following doublet-exclusion (C), only CD5^+^ T cells were included into subsequent analyses (D).
(TIF)

S3 Fig The absence of TCRγδ corresponds with the presence of TCRαβ on CD4^+^CD8α^+^ double-positive (dp) T cells. (A) The expression of TCRαβ and TCRγδ on splenic CD4^+^CD8α^+^ dp T cells was analyzed by flow cytometry. Shown are pseudocolor plots of one representative dog. (B) Proportions of TCRγδ (grey dots) vs. TCRαβ (black dots) expression of mature splenic CD4^+^CD8α^+^ dp T cells were quantified. Each dot represents one individual dog, the horizontal bars indicate mean values.
(TIF)

S4 Fig In lymph nodes, FoxP3^+^CD4^+^CD8α^+^ double-positive (dp) T cells are mainly CD25^high^. (A) Mesenteric lymph node CD4^+^CD8α^+^ dp T cells were analyzed for CD25 and FoxP3 expression. Representative plots show the distribution of FoxP3^+^ cells in CD25^neg^, CD25^dim^ and CD25^high^ subpopulations. The frequency (B) and mean fluorescence intensity (MFI) (C) of FoxP3 expression in CD25^neg^, CD25^dim^, and CD25^high^ CD4^+^CD8α^+^ dp T cells in lymph nodes was quantified. Each symbol represents one individual dog, the horizontal bars indicate median values. Statistical analysis was performed by One-way ANOVA with Dunn’s Multiple Comparison Test (** p < 0.01).
(TIF)

## References

[1] Overgaard NH, Jung J-W, Steptoe RJ, Wells JW. CD4+/CD8+ double-positive T cells: more than just a developmental stage. J Leukoc Biol. 2015; 97: 31–38. 10.1189/jlb.1RU0814-382 25360000

[2] Zuckermann FA. Extrathymic CD4/CD8 double positive T cells. Vet Immunol Immunopathol. 1999; 72: 55–66. 10.1016/S0165-2427(99)00118-X 10614493

[3] Saalmüller A, Reddehase MJ, Bühring HJ, Jonjić S, Koszinowski UH. Simultaneous expression of CD4 and CD8 antigens by a substantial proportion of resting porcine T lymphocytes. Eur J Immunol. 1987; 17: 1297–1301. 10.1002/eji.1830170912 2958295

[4] Pescovitz MD, Lunney JK, Sachs DH. Murine anti-swine T4 and T8 monoclonal antibodies: distribution and effects on proliferative and cytotoxic T cells. J Immunol. 1985; 134: 37–44. 3871107

[5] Blue ML, Daley JF, Levine H, Craig KA, Schlossman SF. Biosynthesis and surface expression of T8 by peripheral blood T4+ cells in vitro. J Immunol. 1986; 137: 1202–1207. 3090142

[6] Akari H. Peripheral blood CD4+CD8+ lymphocytes in cynomolgus monkeys are of resting memory T lineage. International Immunology. 1997; 9: 591–597. 10.1093/intimm/9.4.591 9138020

[7] Takimoto H, Nakamura T, Takeuchi M, Sumi Y, Tanaka T, Nomoto K, et al Age‐associated increase in number of CD4+CD8+ intestinal intraepithelial lymphocytes in rats. Eur J Immunol. 1992; 22: 159–164. 10.1002/eji.1830220124 1370412

[8] Mosley RL, Styre D, Klein JR. CD4+ CD8+ murine intestinal intraepithelial Iymphocytes. Int Immunol. 1990; 2: 361–365. 10.1093/intimm/2.4.361 1980616

[9] Luhtala M, Lassila O, Toivanen P, Vainio O. A novel peripheral CD4+CD8+ T cell population: Inheritance of CD8α expression on CD4+ T cells. Eur J Immunol. 1997; 27: 189–193. 10.1002/eji.1830270128 9022017

[10] Kato M, Watarai S, Nishikawa S, Iwasaki T, Kodama H. A Novel Culture Method of Canine Peripheral Blood Lymphocytes with Concanavalin A and Recombinant Human Interleukin-2 for Adoptive Immunotherapy. J Vet Med Sci. 2007; 69: 481–486. 10.1292/jvms.69.481 17551220

[11] Hoshino Y, Takagi S, Osaki T, Okumura M, Fujinaga T. Phenotypic Analysis and Effects of Sequential Administration of Activated Canine Lymphocytes on Healthy Beagles. J Vet Med Sci. 2008; 70: 581–588. 10.1292/jvms.70.581 18628598

[12] Schütze N, Raue R, Büttner M, Alber G. Inactivated parapoxvirus ovis activates canine blood phagocytes and T lymphocytes. Vet Microbiol. 2009; 137: 260–267. 10.1016/j.vetmic.2009.01.035 19251383

[13] Otani I, Ohta K, Ishikawa A, Yamada T, Ishinazaka T, Ohtaki T, et al Flow Cytometric Analysis of Canine Umbilical Cord Blood Lymphocytes. J Vet Med Sci. 2008; 70: 285–287. 10.1292/jvms.70.285 18388429

[14] Rothe K, Bismarck D, Büttner M, Alber G, Buttlar H von. Canine peripheral blood CD4(+)CD8(+) double-positive Tcell subpopulations exhibit distinct Tcell phenotypes and effector functions. Vet Immunol Immunopathol. 2017; 185: 48–56. 10.1016/j.vetimm.2017.01.005 28242002

[15] Bismarck D, Schütze N, Moore P, Büttner M, Alber G, Buttlar Hv. Canine CD4+CD8+ double positive T cells in peripheral blood have features of activated T cells. Vet Immunol Immunopathol. 2012; 149: 157–166. 10.1016/j.vetimm.2012.06.014 22789871

[16] Bismarck D, Moore PF, Alber G, Buttlar H von. Canine CD4(+)CD8(+) double-positive T cells can develop from CD4(+) and CD8(+) T cells. Vet Immunol Immunopathol. 2014; 162: 72–82. 10.1016/j.vetimm.2014.09.008 25454082

[17] Cheroutre H, Lambolez F. Doubting the TCR coreceptor function of CD8alphaalpha. Immunity. 2008; 28: 149–159. 10.1016/j.immuni.2008.01.005 18275828

[18] Chizzolini C, Parel Y, Luca C de, Tyndall A, Akesson A, Scheja A, et al Systemic sclerosis Th2 cells inhibit collagen production by dermal fibroblasts via membrane-associated tumor necrosis factor alpha. Arthritis Rheum. 2003; 48: 2593–2604. 10.1002/art.11129 13130479

[19] Kitchen SG, Jones NR, LaForge S, Whitmire JK, Vu B-A, Galic Z, et al CD4 on CD8(+) T cells directly enhances effector function and is a target for HIV infection. Proc Natl Acad Sci U S A. 2004; 101: 8727–8732. 10.1073/pnas.0401500101 15173593 PMC423263

[20] Sarrabayrouse G, Bossard C, Chauvin J-M, Jarry A, Meurette G, Quévrain E, et al CD4CD8αα lymphocytes, a novel human regulatory T cell subset induced by colonic bacteria and deficient in patients with inflammatory bowel disease. PLoS Biol. 2014; 12: e1001833 10.1371/journal.pbio.1001833 24714093 PMC3979654

[21] Bang K, Lund M, Wu K, Mogensen SC, Thestrup-Pedersen K. CD4+ CD8+ (thymocyte-like) T lymphocytes present in blood and skin from patients with atopic dermatitis suggest immune dysregulation. Br J Dermatol. 2001; 144: 1140–1147. 10.1046/j.1365-2133.2001.04223.x 11422033

[22] Desfrançois J, Derré L, Corvaisier M, Le Mével B, Catros V, Jotereau F, et al Increased frequency of nonconventional double positive CD4CD8 alphabeta T cells in human breast pleural effusions. Int J Cancer. 2009; 125: 374–380. 10.1002/ijc.24366 19358272

[23] McGill JL, Wang Y, Ganta CK, Boorgula GDY, Ganta RR. Antigen-Specific CD4+CD8+ Double-Positive T Cells Are Increased in the Blood and Spleen During Ehrlichia chaffeensis Infection in the Canine Host. Front Immunol. 2018; 9: 1585 10.3389/fimmu.2018.01585 30050533 PMC6050357

[24] Alexandre-Pires G, Brito MTV de, Algueró C, Martins C, Rodrigues OR, da Fonseca IP, et al Canine leishmaniasis. Immunophenotypic profile of leukocytes in different compartments of symptomatic, asymptomatic and treated dogs. Vet Immunol Immunopathol. 2010; 137: 275–283. 10.1016/j.vetimm.2010.06.007 20615553

[25] Solano-Aguilar GI, Vengroski KG, Beshah E, Lunney JK. Isolation and purification of lymphocyte subsets from gut-associated lymphoid tissue in neonatal swine. J Immunol Methods. 2000; 241: 185–199. 10.1016/S0022-1759(00)00209-X 10915860

[26] Mizuno T, Suzuki R, Umeki S, Okuda M. Crossreactivity of Antibodies to Canine CD25 and Foxp3 and Identification of Canine CD4+CD25 +Foxp3+ Cells in Canine Peripheral Blood. J Vet Med Sci. 2009; 71: 1561–1568. 10.1292/jvms.001561 20046022

[27] Junginger J, Schwittlick U, Lemensieck F, Nolte I, Hewicker-Trautwein M. Immunohistochemical investigation of Foxp3 expression in the intestine in healthy and diseased dogs. Vet Res. 2012; 43: 23 10.1186/1297-9716-43-23 22440243 PMC3364872

[28] Olivares-Villagómez D, van Kaer L. Intestinal Intraepithelial Lymphocytes: Sentinels of the Mucosal Barrier. Trends Immunol. 2018; 39: 264–275. 10.1016/j.it.2017.11.003 29221933 PMC8056148

[29] Luckschander N, Pfammatter NS, Sidler D, Jakob S, Burgener IA, Moore PF, et al Phenotyping, functional characterization, and developmental changes in canine intestinal intraepithelial lymphocytes. Vet Res. 2009; 40: 58 10.1051/vetres/2009042 19631032

[30] Godfrey DI, Zlotnik A. Control points in early T-cell development. Immunol Today. 1993; 14: 547–553. 10.1016/0167-5699(93)90186-O 7903854

[31] He YW, Nakajima H, Leonard WJ, Adkins B, Malek TR. The common gamma-chain of cytokine receptors regulates intrathymic T cell development at multiple stages. J Immunol. 1997; 158: 2592–2599. 9058791

[32] Sinkora M, Sinkora J, Rehakova Z, Butler JE. Early Ontogeny of Thymocytes in Pigs: Sequential Colonization of the Thymus by T Cell Progenitors. J Immunol. 2000; 165: 1832–1839. 10.4049/jimmunol.165.4.1832 10925261

[33] Fontenot JD, Gavin MA, Rudensky AY. Foxp3 programs the development and function of CD4+CD25+ regulatory T cells. Nat Immunol. 2003; 4: 330–336. 10.1038/ni904 12612578

[34] Roederer M. How many events is enough? Are you positive. Cytometry A. 2008; 73: 384–385. 10.1002/cyto.a.20549 18307257

[35] Mayassi T, Jabri B. Human intraepithelial lymphocytes. Mucosal Immunol. 2018; 11: 1281–1289. 10.1038/s41385-018-0016-5 29674648 PMC6178824

[36] Mucida D, Husain MM, Muroi S, van Wijk F, Shinnakasu R, Naoe Y, et al Transcriptional reprogramming of mature CD4⁺ helper T cells generates distinct MHC class II-restricted cytotoxic T lymphocytes. Nat Immunol. 2013; 14: 281–289. 10.1038/ni.2523 23334788 PMC3581083

[37] Takeuchi A, Saito T. CD4 CTL, a Cytotoxic Subset of CD4+ T Cells, Their Differentiation and Function. Front Immunol. 2017; 8: 194 10.3389/fimmu.2017.00194 28280496 PMC5321676

[38] Rütgen BC, König R, Hammer SE, Groiss S, Saalmüller A, Schwendenwein I. Composition of lymphocyte subpopulations in normal canine lymph nodes. Vet Clin Pathol. 2015; 44: 58–69. 10.1111/vcp.12221 25512102

[39] Sonea IM, Jergens AE, Sacco RE, Niyo Y, Merten E, Kauffman LK, et al Flow cytometric analysis of colonic and small intestinal mucosal lymphocytes obtained by endoscopic biopsy in the healthy dog. Vet Immunol Immunopathol. 2000; 77: 103–119. 11068069 10.1016/s0165-2427(00)00230-0

[40] Kern P, Hussey RE, Spoerl R, Reinherz EL, Chang H-C. Expression, Purification, and Functional Analysis of Murine Ectodomain Fragments of CD8αα and CD8αβ Dimers. J Biol Chem. 1999; 274: 27237–27243. 10.1074/jbc.274.38.27237 10480942

[41] McNicol A‐M, Bendle G, Holler A, Matjeka T, Dalton E, Rettig L, et al CD8α/α homodimers fail to function as co‐receptor for a CD8‐dependent TCR. Eur J Immunol. 2007; 37: 1634–1641. 10.1002/eji.200636900 17506031

[42] Cawthon AG, Lu H, Alexander-Miller MA. Peptide Requirement for CTL Activation Reflects the Sensitivity to CD3 Engagement: Correlation with CD8αβ Versus CD8αα Expression. J Immunol. 2001; 167: 2577–2584. 10.4049/jimmunol.167.5.2577 11509598

[43] Spolski R, Li P, Leonard WJ. Biology and regulation of IL-2: from molecular mechanisms to human therapy. Nat Rev Immunol. 2018; 18: 648–659. 10.1038/s41577-018-0046-y 30089912

[44] Lefrancois L. Phenotypic complexity of intraepithelial lymphocytes of the small intestine. J Immunol. 1991; 147: 1746–1751. 1716278

[45] Cheroutre H. Starting at the beginning: new perspectives on the biology of mucosal T cells. Annu Rev Immunol. 2004; 22: 217–246. 10.1146/annurev.immunol.22.012703.104522 15032579

[46] Morrissey PJ, Charrier K, Horovitz DA, Fletcher FA, Watson JD. Analysis of the intra-epithelial lymphocyte compartment in SCID mice that received co-isogenic CD4+ T cells. Evidence that mature post-thymic CD4+ T cells can be induced to express CD8 alpha in vivo. J Immunol. 1995; 154: 2678–2686. 7876540

[47] Aranda R, Sydora BC, McAllister PL, Binder SW, Yang HY, Targan SR, et al Analysis of intestinal lymphocytes in mouse colitis mediated by transfer of CD4+, CD45RBhigh T cells to SCID recipients. J Immunol. 1997; 158: 3464–3473. 9120308

[48] Reis BS, Rogoz A, Costa-Pinto FA, Taniuchi I, Mucida D. Mutual expression of the transcription factors Runx3 and ThPOK regulates intestinal CD4(+) T cell immunity. Nat Immunol. 2013; 14: 271–280. 10.1038/ni.2518 23334789 PMC3804366

[49] Oliveira LdM, Teixeira FME, Sato MN. Impact of Retinoic Acid on Immune Cells and Inflammatory Diseases. Mediators Inflamm. 2018; 2018: 3067126 10.1155/2018/3067126 30158832 PMC6109577

[50] Reboldi A, Cyster JG. Peyer's patches: organizing B-cell responses at the intestinal frontier. Immunol Rev. 2016; 271: 230–245. 10.1111/imr.12400 27088918 PMC4835804

[51] Iwata M, Hirakiyama A, Eshima Y, Kagechika H, Kato C, Song S-Y. Retinoic acid imprints gut-homing specificity on T cells. Immunity. 2004; 21: 527–538. 10.1016/j.immuni.2004.08.011 15485630

[52] Zuckermann FA, Gaskins HR. Distribution of porcine CD4/CD8 double-positive T lymphocytes in mucosa-associated lymphoid tissues. Immunology. 1996; 87: 493–499. 10.1046/j.1365-2567.1996.494570.x 8778039 PMC1384122

[53] Das G, Augustine MM, Das J, Bottomly K, Ray P, Ray A. An important regulatory role for CD4+CD8 alpha alpha T cells in the intestinal epithelial layer in the prevention of inflammatory bowel disease. Proc Natl Acad Sci U S A. 2003; 100: 5324–5329. 10.1073/pnas.0831037100 12695566 PMC154344

[54] Hillemeyer P, White MD, Pascual DW. Development of a transient CD4+CD8+ T cell subset in the cervical lymph nodes following intratracheal instillation with an adenovirus vector. Cell Immunol. 2002; 215: 173–185. 10.1016/S0008-8749(02)00024-2 12202154

[55] Abuzakouk M, Carton J, Feighery C, OʼDonoghue DP, Weir DG, OʼFarrelly C. CD4+CD8+ and CD8α+β- T lymphocytes in human small intestinal lamina propria. Eur J Gastroenterol Hepatol. 1998; 10: 325–330. 10.1097/00042737-199804000-00009 9855049

[56] Käser T, Gerner W, Hammer SE, Patzl M, Saalmüller A. Detection of Foxp3 protein expression in porcine T lymphocytes. Vet Immunol Immunopathol. 2008; 125: 92–101. 10.1016/j.vetimm.2008.05.007 18565594

[57] van Kaer L, Rabacal WAS, Scott Algood HM, Parekh VV, Olivares-Villagómez D. In vitro induction of regulatory CD4+CD8α+ T cells by TGF-β, IL-7 and IFN-γ. PLoS ONE. 2013; 8: e67821 10.1371/journal.pone.0067821 23844100 PMC3701067

[58] Sujino T, London M, van Hoytema Konijnenburg DP, Rendon T, Buch T, Silva HM, et al Tissue adaptation of regulatory and intraepithelial CD4⁺ T cells controls gut inflammation. Science. 2016; 352: 1581–1586. 10.1126/science.aaf3892 27256884 PMC4968079

