# Supplementary figures and images for: Canine tissue-associated CD4+CD8α+ double-positive T cells are an activated T cell subpopulation with heterogeneous functional potential
Source: PLoS One. 2019 Mar 13;14(3):e0213597. doi: 10.1371/journal.pone.0213597 (PMC6415905; doi:10.1371/journal.pone.0213597)

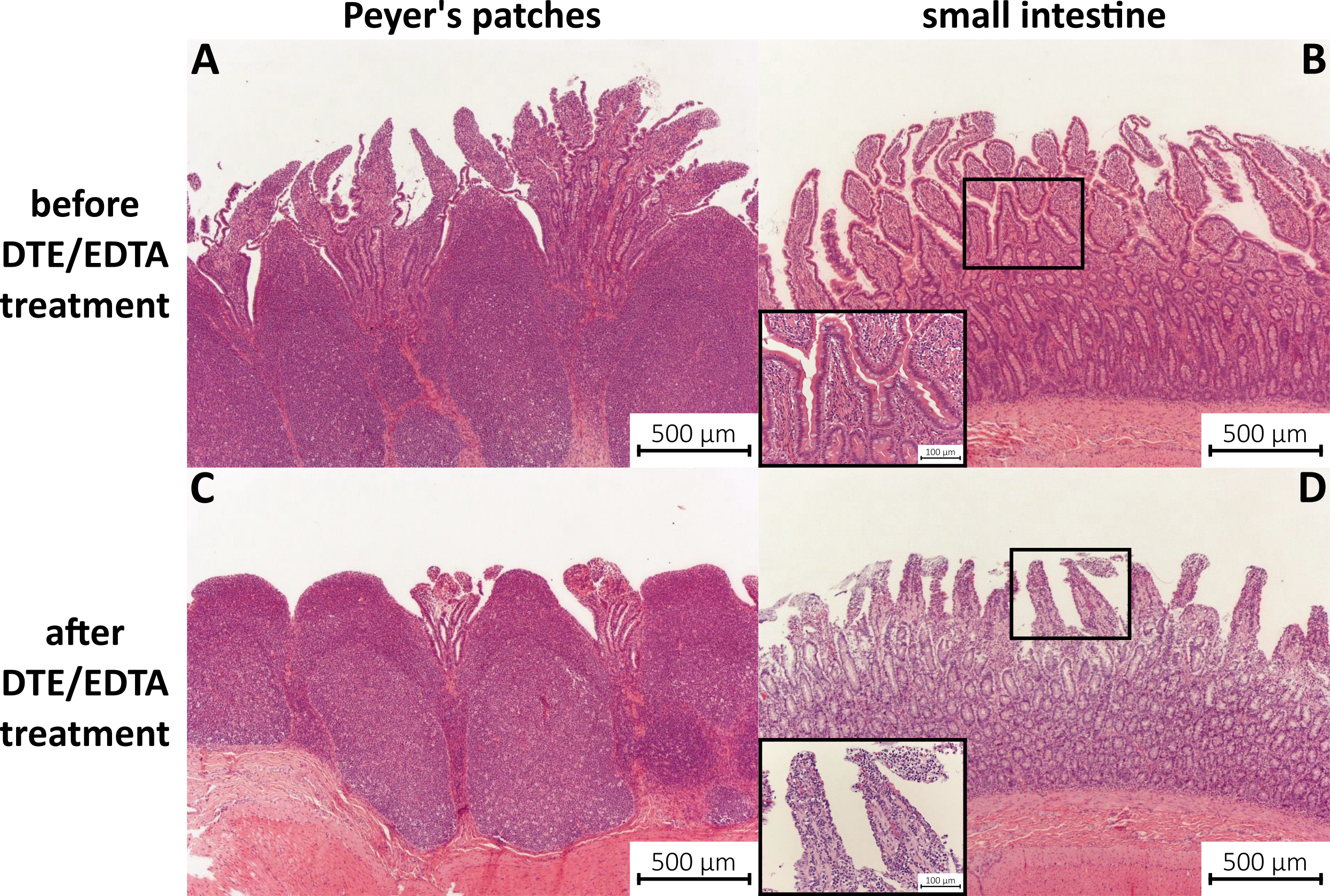

Supplement: S1 Fig — Representative H&E-stained sections of Peyer’s patches (A) and small intestine (B) before DTE/EDTA treatment are shown. Normal villous architecture with intact epithelium is visible. After DTE/EDTA treatment, the epithelial layer is removed from Peyer’s patches (C), and from the villi (D), respectively. For the latter this is displayed at higher magnifications in the insets (compare B + D). (TIF) [file pone.0213597.s001.tif]

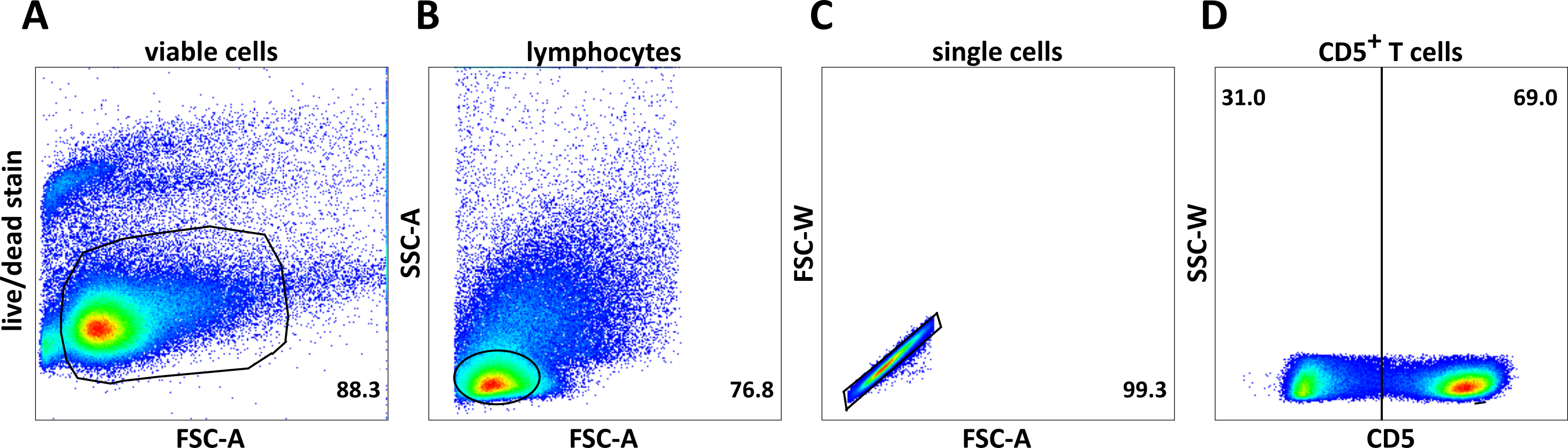

Supplement: S2 Fig — Representative pseudocolor plots of Peyer’s patches are depicted to show the general gating strategy. After exclusion of dead cells (A), gating on lymphocytes was performed according to their forward and side scattering (FSC/SSC) properties (B). Following doublet-exclusion (C), only CD5+ T cells were included into subsequent analyses (D). (TIF) [file pone.0213597.s002.tif]

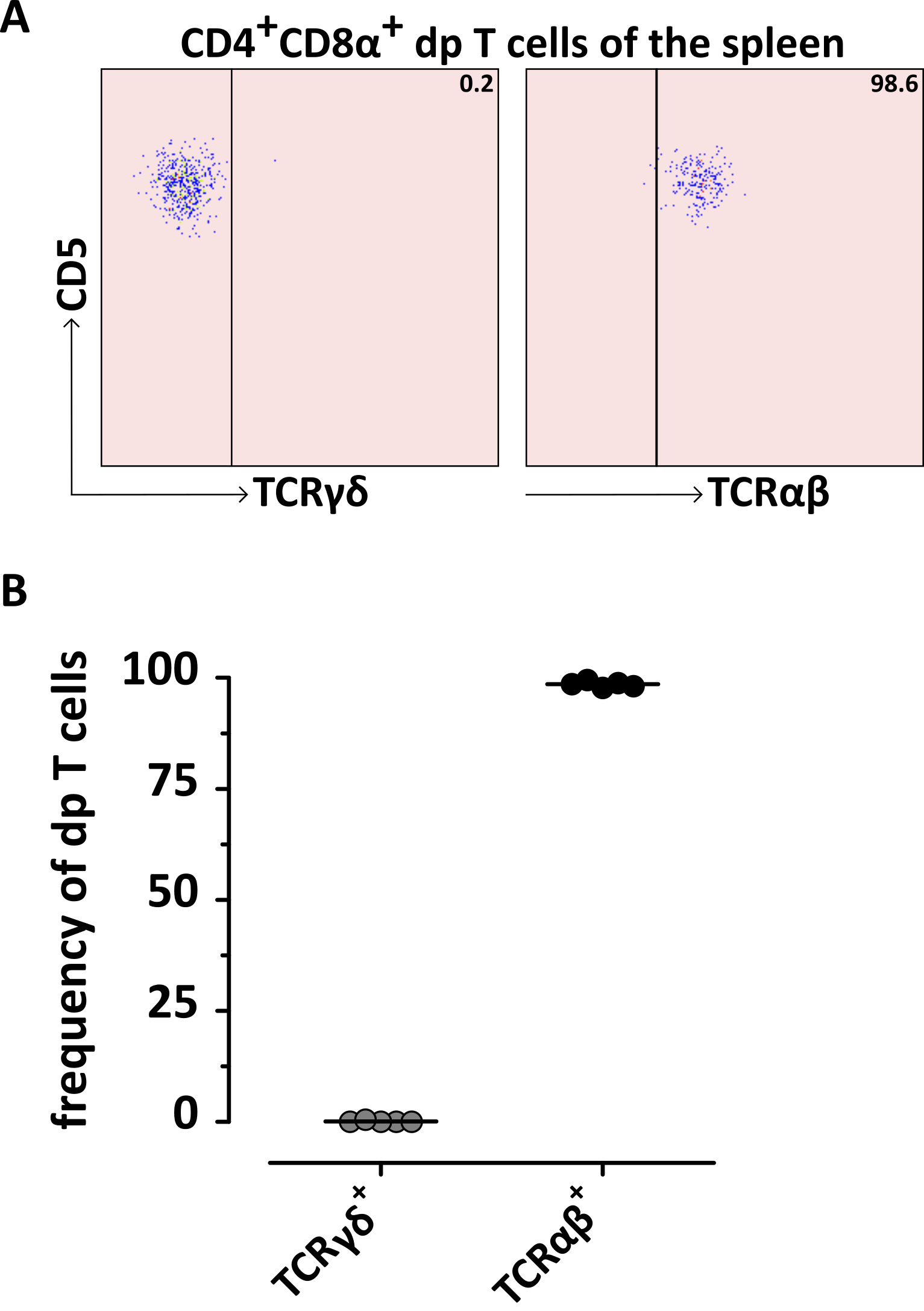

Supplement: S3 Fig — (A) The expression of TCRαβ and TCRγδ on splenic CD4+CD8α+ dp T cells was analyzed by flow cytometry. Shown are pseudocolor plots of one representative dog. (B) Proportions of TCRγδ (grey dots) vs. TCRαβ (black dots) expression of mature splenic CD4+CD8α+ dp T cells were quantified. Each dot represents one individual dog, the horizontal bars indicate mean values. (TIF) [file pone.0213597.s003.tif]

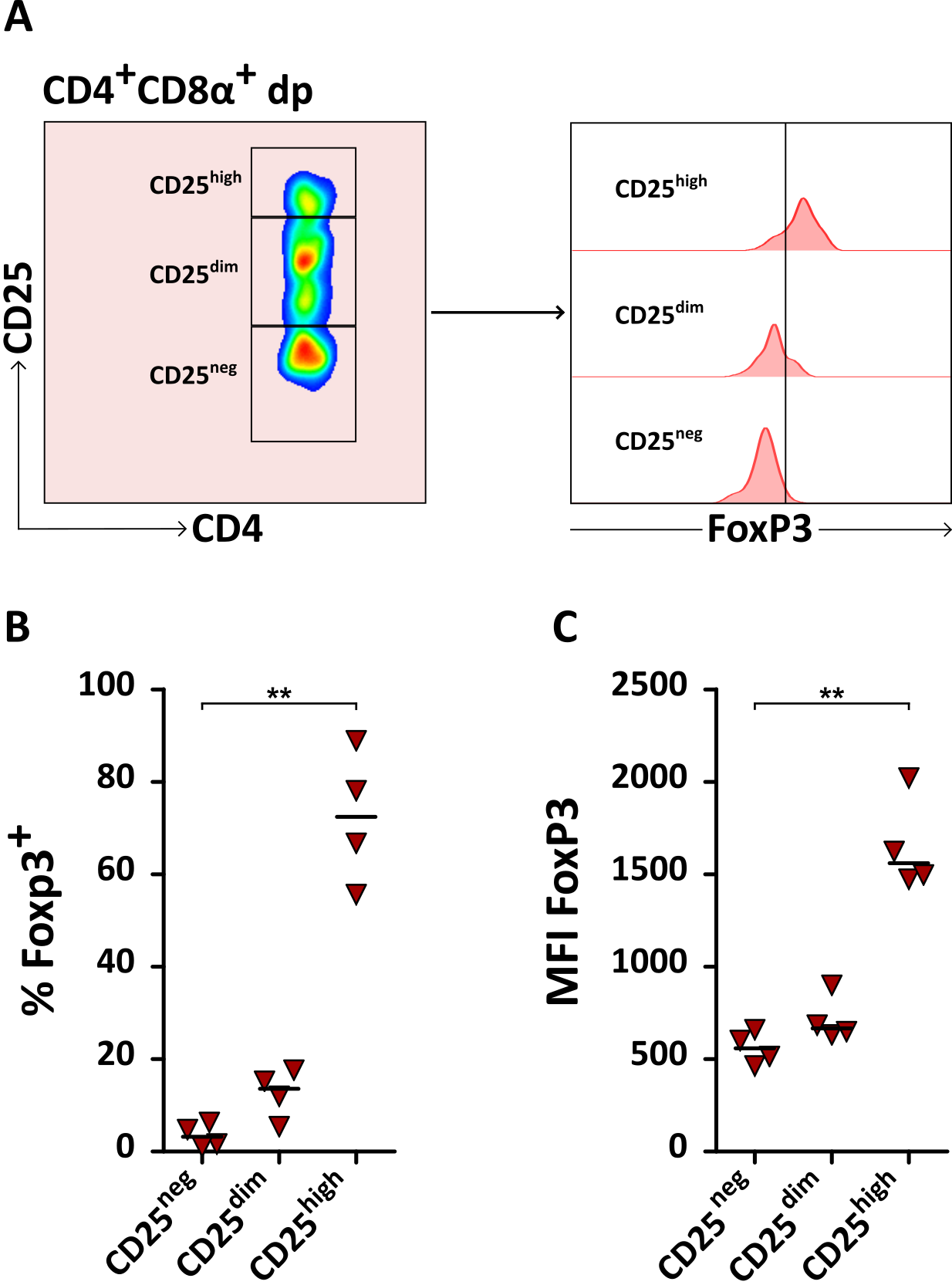

Supplement: S4 Fig — (A) Mesenteric lymph node CD4+CD8α+ dp T cells were analyzed for CD25 and FoxP3 expression. Representative plots show the distribution of FoxP3+ cells in CD25neg, CD25dim and CD25high subpopulations. The frequency (B) and mean fluorescence intensity (MFI) (C) of FoxP3 expression in CD25neg, CD25dim, and CD25high CD4+CD8α+ dp T cells in lymph nodes was quantified. Each symbol represents one individual dog, the horizontal bars indicate median values. Statistical analysis was performed by One-way ANOVA with Dunn’s Multiple Comparison Test (** p < 0.01). (TIF) [file pone.0213597.s004.tif]
